# Supplementary material for: Uncovering the Resistance Mechanisms in Extended-Drug-Resistant Pseudomonas aeruginosa Clinical Isolates: Insights from Gene Expression and Phenotypic Tests
Source: Microorganisms. 2023 Aug 31;11(9):2211. doi: 10.3390/microorganisms11092211 (PMC10535578; doi:10.3390/microorganisms11092211)
Supplement: Supplementary file 1 [file microorganisms-11-02211-s001.zip › microorganisms-2542208-SI.pdf]

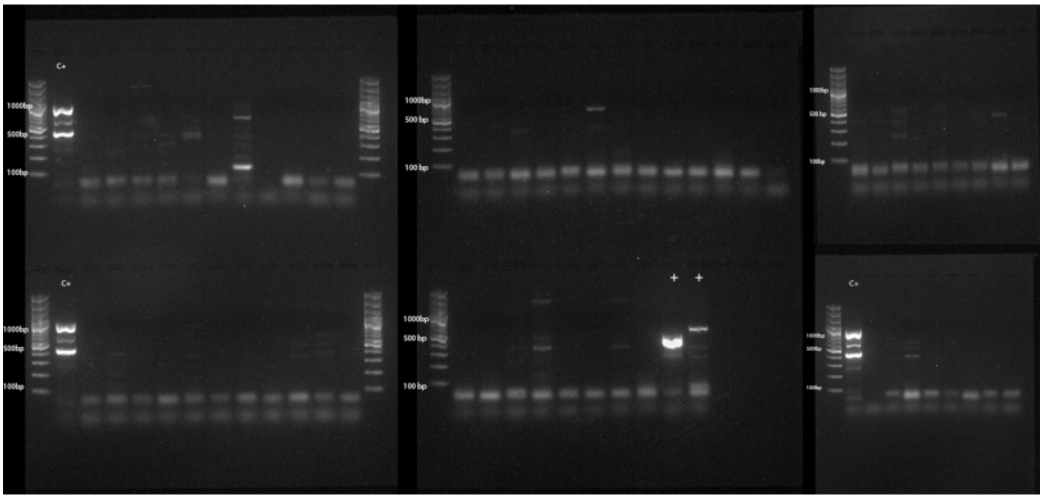

Figure S1. Gel electrophoresis following triplex PCR for *bla*$_{KPC}$, *bla*$_{NDM}$, *bla*$_{OXA48\text{-}like}$; showing positive bands for *bla*$_{OXA48\text{-}like}$ and *bla*$_{NDM}$ genes for the isolates 59 and 60 are marked with "+", corresponding to approximately 438 bp, respectively 621 bp; (C+) positive control.

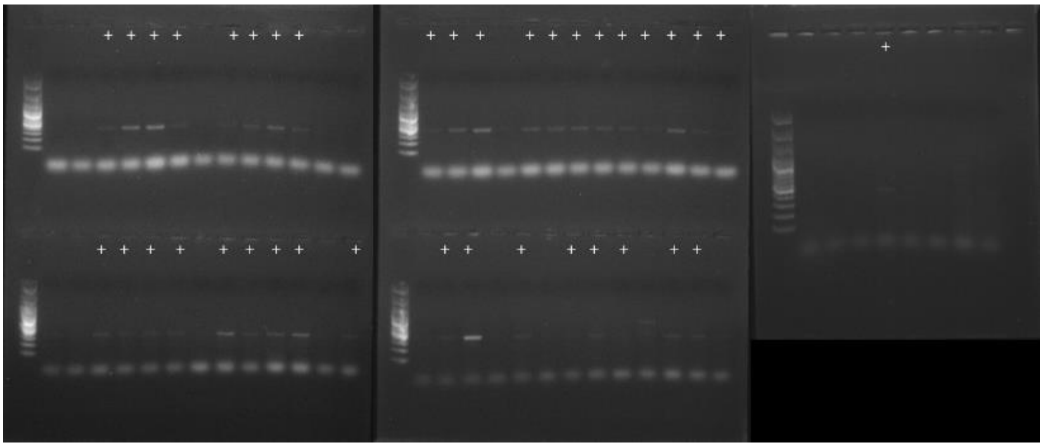

Figure S2. Gel electrophoresis following PCR for *bla*$_{GES\text{-}2}$;; positive strains are marked with "+", corresponding to 371 bp.

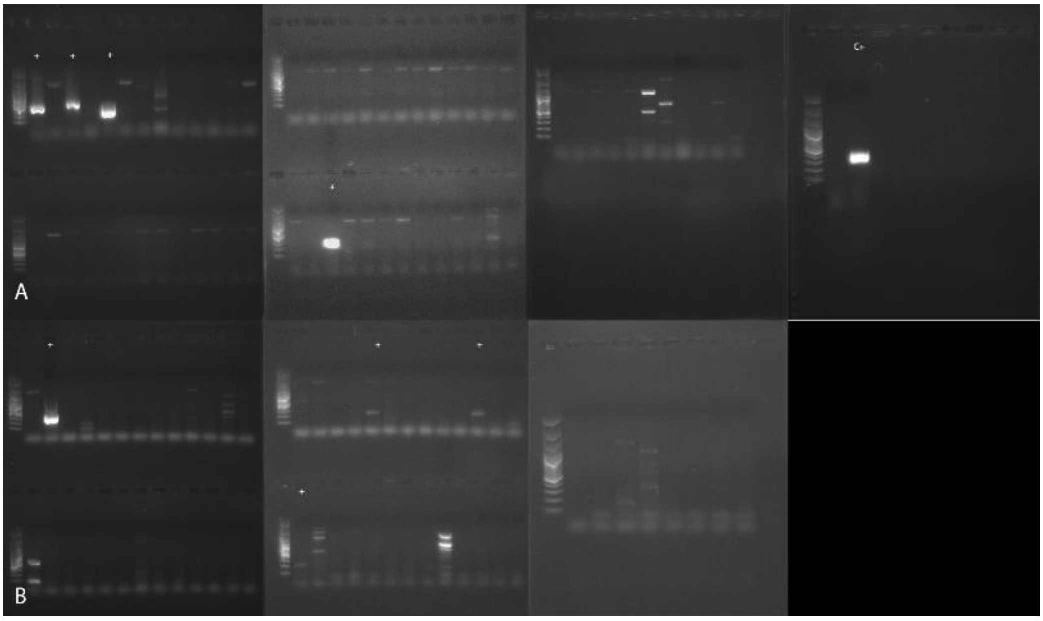

Figure S3. Gel electrophoresis following PCR for *bla*$_{VIM}$ (A) and *bla*$_{SPM}$ (B); positive strains are marked with "+", corresponding to 271 bp for *bla*$_{SPM}$ and 390 for *bla*$_{VIM}$.
